# Spectral separation of optical spin based on antisymmetric Fano resonances

**DOI:** 10.1038/srep16585

**Published:** 2015-11-12

**Authors:** Xianji Piao, Sunkyu Yu, Jiho Hong, Namkyoo Park

**Affiliations:** 1 Photonic Systems Laboratory, School of EECS, Seoul National University, Seoul 151-744, Korea

## Abstract

We propose a route to the spectral separation of optical spin angular momentum based on spin-dependent Fano resonances with antisymmetric spectral profiles. By developing a spin-form coupled mode theory for chiral materials, the origin of antisymmetric Fano spectra is clarified in terms of the opposite temporal phase shift for each spin, which is the result of counter-rotating spin eigenvectors. An analytical expression of a spin-density Fano parameter is derived to enable quantitative analysis of the Fano-induced spin separation in the spectral domain. As an application, we demonstrate optical spin switching utilizing the extreme spectral sensitivity of the spin-density reversal. Our result paves a path toward the conservative spectral separation of spins without any need of the magneto-optical effect or circular dichroism, achieving excellent purity in spin density superior to conventional approaches based on circular dichroism.

Optical spin angular momentum (SAM) in relation to the handedness of photons^1^ is a topic that has received significant attention. The SAM of light is typically achieved with materials which are distinguishable from their mirror images i.e. optical chiral materials^2^,^3^,^4^,^5^,^6^,^7^,^8^,^9^,^10^. Overcoming the restrictions in natural chiral materials, the field of optical chirality is now entering into a new regime of artificial chirality, with the progress in fabrication technology. Artificially enhanced chiral interactions^4^,^5^,^6^,^7^,^8^,^11^,^12^,^13^ using subwavelength structures have been demonstrated not only for quantum-analogical interactions such as spin-orbit coupling^14^,^15^, but also for a variety of applications, including enantiomer sensing^12^,^13^, negative refraction^16^,^17^,^18^, and topological bandgaps^19^,^20^. Approaches for notable artificial structures also include nature-mimetic 3-dimensional metamaterials of giant chirality^4^,^5^,^6^,^16^,^17^, the mixing of electric and magnetic responses for oblique incidences to anisotropic metasurfaces^21^,^22^,^23^, and 2-dimensional meta-films using the overlap of electric- and magnetic- dipoles^7^,^8^,^18^ or non-Hermitian electric dipoles^24^.

In the context of the achievement of optical SAM, chiral materials alone do not establish the sufficient condition, in spite of their spin-dependent wavevectors (Fig. 1a). This is due to the spin-independent wave impedance of chiral materials, which leads to equal SAM scattering intensity^2^,^3^; in contrast to gyrotropic materials with spin-dependent *wavevectors and impedances*^3^,^25^. An alternative route toward optical SAM in the absence of gyrotropic impedance can be made using circular dichroism^2^,^3^,^7^,^8^,^18^,^24^,^26^,^27^ to selectively ‘*annihilate*’ spins (Fig. 1b), yet at the expense of inherent dissipation and the consequent degradation of the quality factor in the system. To achieve *conservative* and high-*Q* response in optical SAM with nonmagnetic materials, the spectral ‘*separation*’ of spin can be envisaged (as in Fig. 1c), yet has not been sought or demonstrated.

In this paper, we propose and demonstrate the nonmagnetic conservative ‘*separation*’ of optical SAM, derived from the difference of two antisymmetric and spectrally separated Fano resonance spectra (Fig. 1c). Understanding that Fano resonances involve inherently asymmetric spectral profiles with shifted resonances^28^,^29^,^30^,^31^, it will be shown that, upon the opposite shift of Fano spectral pole for different handedness of photon, the spectral separation of spin and consequently the nonzero value of SAM can be achieved. As an implementation platform, we analyze a chiral resonator with a pair of highly-birefringent mirrors, which provide *x*- and *y*- polarization dependent scattering pathways for the Fano interference. By developing a temporal coupled mode theory (CMT)^32^,^33^ for the Fano chiral resonator, we then prove that the handedness of the spin eigenvector is projected onto the temporal domain as an opposite temporal shift, thereby leading to an antisymmetric Fano response in the spectral domain. The spin-density Fano parameter in relation to the material chirality and mirror birefringence is also derived for the quantitative control of optical SAM. Finally, as an application, we propose ‘optical spin switching’ and the practical design based on indefinite metamaterial mirrors, with experimentally accessible material parameters.

## Results

### Realization of spin-dependent Fano resonances

**Figure 2:**
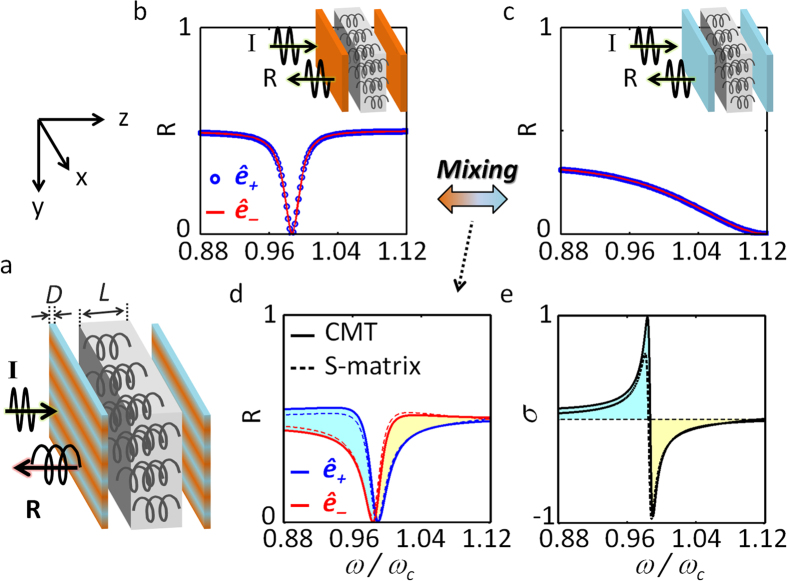
Spectral separation of optical SAM based on antisymmetric Fano resonances. (**a**) Proposed chiral resonator for SAM separation. A layer of a chiral medium (*L* = 0.29*λ*_*c*_ where *λ*_*c*_ = 2*πc*/*ω*_*c*_ and *ω*_*c*_ is the normalization frequency, *ε*_*chiral*_ = 9, *χ* = 0.05) is enclosed by a pair of birefringent mirrors (*ε*_*x*_ = *ε*_*metal*_ = −80, *ε*_*y*_ = *ε*_*dielec*_ = 2.25, *D* = *λ*_*c*_/60). Reflection spectra of *ê*_+_ (blue) and *ê*_−_ (red) spin modes with (**b**) metallic mirrors (*ε*_*x*_ = *ε*_*y*_ = *ε*_*metal*_), (**c**) dielectric mirrors (*ε*_*x*_ = *ε*_*y*_ = *ε*_*dielec*_), and (**d**) highly-birefringent mirrors (*ε*_*x*_ = *ε*_*metal*_, *ε*_*y*_ = *ε*_*dielec*_). (**e**) Spectra of SAM density *σ* (±1 denotes pure *ê*_±_) with birefringent mirrors. The calculations are based on both CMT (solid) and a scattering matrix (dashed). The CMT fitting values for the resonance (*ω*_*x*_ = 0.987*ω*_*c*_ and *ω*_*y*_ = 0.571*ω*_*c*_) and *Q*-factors (*Q*_*x*_ = 100 and *Q*_*y*_ = 12) for each mode (Fig. 2b,c) were obtained from the scattering matrix results.

Figure 2a shows a schematic diagram of the proposed structure for the Fano-resonant separation of optical SAM. A Fabry-Perot resonator is constructed with a chiral material sandwiched between a pair of birefringent (*ε*_*x*_, *ε*_*y*_) mirrors. To clearly isolate the physics of Fano-induced optical SAM from circular dichroism, for the moment we use dielectric constants of real values (inclusion of material loss will be treated later, and also in Supplementary Note 1 and 2). For the special case with isotropic mirrors of *ε*_*x*_ = *ε*_*y*_, the response of the resonator per the incidence of *x*-polarized plane wave is calculated with CMT (see refs 29,30 and Eqs. (1, 2, 3) for the spin-form CMT) and a scattering matrix^34^, as shown in Fig. 2b,c (with *ε*_*x*,*y*_ = *ε*_*metal*_ = −80 and *ε*_*x*,*y*_ = *ε*_*dielec*_ = 2.25 respectively. please see Supplementary Notes 1 and 2 for its realization). With perfect overlap between the reflection spectra of the *ê*_±_ spin modes, there is no optical SAM when *ε*_*x*_ = *ε*_*y*_, as expected in refs 2,3.

For comparison, Fig. 2d shows the reflection spectra of the resonator, with the highly *birefringent* film (*ε*_*x*_ = *ε*_*metal*_ ≪ 0 < *ε*_*y*_ = *ε*_*dielec*_). In stark contrast to the case of *ε*_*x*_ = *ε*_*y*_, the spectral separation of the *ê*_±_ spin mode (*ê*_+_ in blue and *ê*_−_ in red) and the separation of optical SAM (light-blue and yellow regions, Fig. 2d) is evident. Figure 2e shows the calculated spin density *σ* = (*R*_*ê*−_ − *R*_*ê*+_)/(*R*_*ê*−_ + *R*_*ê*+_), where *R*_*ê*±_ is the reflectance of the *ê*_±_ component, and *σ* = ±1 represents the pure spin state *ê*_±_. Compared with the case of circular dichroism (*σ* ~ 0.5)^27^, a much larger spin density value *σ* ~ 0.998, close to the pure spin state, is achieved from the difference of two narrowband and antisymmetric Fano profiles. Meanwhile, it is clear that the emergence of Fano resonances for each spin state *σ* = ±1 is the result of the mixing between the narrowband (Fig. 2b, through *x*-axis in mirrors) and broadband (Fig. 2c, through *y*-axis in mirrors) scattering pathways (*ε*_*x*_ = *ε*_*metal*_ ≪ 0 < *ε*_*y*_ = *ε*_*dielec*_); the underlying physics of the opposite, antisymmetric shift of the Fano spectral pole in relation to the handedness of the spin needs to be further elaborated, as detailed in the later section (see Supplementary Note 3 for the dependency on the state of linear polarizations).

**Figure 3:**
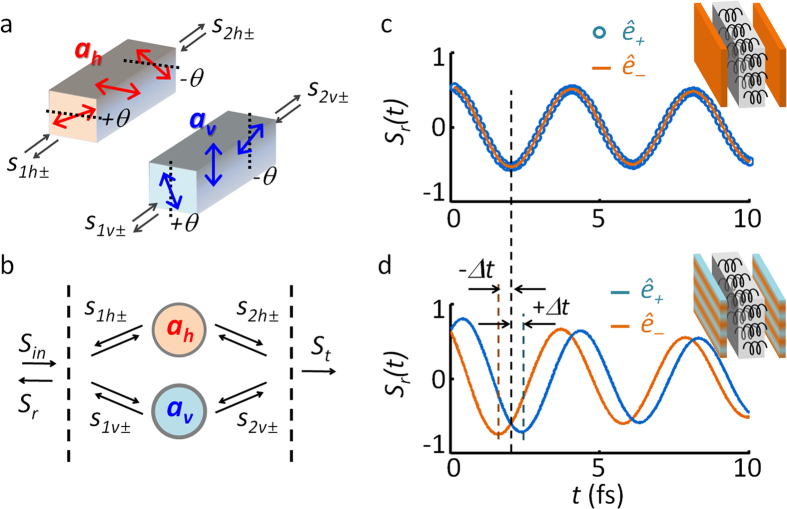
Coupled mode analysis for Fano-chiral systems. (**a**) Representation of a chiral resonator in linear basis *h* and *v*, including natural optical rotation (horizontal and vertical in the middle of the resonator and rotated by ±*θ* at the two interfaces). *S*_*1*(*2*)*h*(*v*)±_ denotes the respective polarization component of incident wave at the interface. (**b**) The CMT model of the chiral resonator illustrated in Fig. 2a, with *S*_*in*_: incidence, *S*_*r*_: reflection, and *S*_*t*_: transmission. (**c**,**d**) Impulse responses of the resonator for different spins (*S*_*1x*_^*in*^ = *δ*(*t*), *ê*_+_: blue, *ê*_−_: red), with (**c**) *ε*_*x*_ = *ε*_*y*_ = *ε*_*metal*_ and (**d**) *ε*_*x*_ = *ε*_*metal*_, *ε*_*y*_ = *ε*_*dielec*_. The dashed lines indicate temporal shifts of *Δt* = 2*θ*/*ω*_c_, obtained for *ê*_±_ respectively. All of the results are calculated using the temporal CMT equation (Eq. (3)).

To investigate the origin of the observed spin-dependent antisymmetric Fano responses, we first need to develop a temporal CMT for chiral resonances. Considering the natural optical rotation 2*θ* (*θ* = *ωχL*_*eff*_/2*c*, *χ*: normalized chirality, *L*_*eff*_: effective path, *c*: speed of light)^2^,^3^ inside the resonator, we introduce the ‘rotated’ coordinates (*h*- and *v*-axes in Fig. 3a) for a chiral medium. The chiral resonant mode can then be decomposed into two linear resonant modes (*a*_*h*_, *a*_*v*_), orthogonal to each other and having modal decay times of *τ*_*h*_ and *τ*_*v*_ respectively (Fig. 3a). Because the *h*- and *v*-axes are rotated by *θ* from the middle of the cavity to the mirror, we obtain 1/*τ*_*h*_ = cos^2^*θ*/*τ*_*x*_ + sin^2^*θ*/*τ*_*y*_ and 1/*τ*_*v*_ = sin^2^*θ*/*τ*_*x*_ + cos^2^*θ*/*τ*_*y*_, where *τ*_*x*_ and *τ*_*y*_ are the decay times for each birefringence axis (*τ*_*x*_ ≠ *τ*_*y*_ when *ε*_*x*_ ≠ *ε*_*y*_). The CMT equation in *h*- and *v*- coordinates then becomes


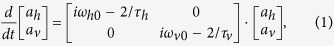


where *ω*_*h0*_ (or *ω*_*v*0_) is the resonant frequency of the *a*_*h*_ (or *a*_*v*_) mode. Upon the incident of *x*- and *y*-polarized waves (*S*_*1x*+_, *S*_*1y*+_) to the resonator (Fig. 3b), their couplings to resonance modes (*a*_*h*_, *a*_*v*_) are thus written as


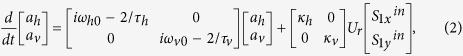


where *U*_*r*_ is the rotation matrix of [cos*θ*, sin*θ*; −sin*θ*, cos*θ*] and *κ*_*h*,*v*_ = (2/*τ*_*h*,*v*_)^1/2^ is the excitation coupling coefficient to the resonator^32^,^33^.

In terms of the optical SAM, it is convenient to use a spin-basis representation of Eq. (2). Taking the spin form of 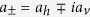 and 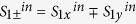, we then achieve the spin-form CMT, upon the incident wave of ***S***_*in*_ = [*S*_*1*+_^*in*^;*S*_*1*−_^*in*^] as follows:


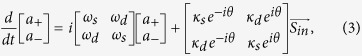


where *ω*_*s*_ = (*ω*_*h0*_ + *ω*_*v0*_)/2 + *i* · (1/*τ*_*h*_ + 1/*τ*_*v*_), *ω*_*d*_ = (*ω*_*h0*_ − *ω*_*v0*_)/2 + *i* · (1/*τ*_*h*_ − 1/*τ*_*v*_), *κ*_*s*_ = (*κ*_*h*_ + *κ*_*v*_)/2, and *κ*_*d*_ = (*κ*_*h*_ − *κ*_*v*_)/2. We emphasize that this spin-form CMT (Eq. (3)) clearly reveals the underlying physics of the spin-dependent Fano responses. First, mixing between spin modes *a*_+_ and *a*_−_ through nonzero *ω*_*d*_ and *κ*_*d*_ arises when *τ*_*x*_ ≠ *τ*_*y*_ (the birefringent mirror case), breaking the spectral degeneracy of the spin modes. Second, the incident waves undergo phase evolutions through *κ*_*s*_*e*^±*iθ*^, in *opposite* directions for *ê*_+_ and *ê*_−_ spins, thus deriving the antisymmetric Fano response for opposite spins.

The temporal interpretation of Fano dynamics^35^, for the impulse response of Eq. (3) with *S*_*1x*_^*in*^ = *δ*(*t*), further elucidates the origin of antisymmetric Fano resonances for opposite spin states. Figure 3c,d show the impulse responses of the chiral resonators, having isotropic and birefringence mirrors respectively. The temporal phase shift corresponding to Fano resonance^35^, especially in the opposite direction for the *ê*_+_ and *ê*_−_ spin modes, exposes only when both conditions of *θ* ≠ *0* (chiral medium) and *τ*_*h*_ ≠ *τ*_*v*_ (birefringent mirrors) are met at the same time. In detail, the phase shift for each spin *ê*_+_ and *ê*_−_ takes the form of time-leading and lagging (*Δt* = 2*θ*/*ω*_c_), from the different scattering paths *κ*_*d*_*e*^±*iθ*^ (Eq. (3)). In the spectral representation these temporal shifts correspond to shifts in the Fano resonant poles^35^ in opposite directions, and thus lead to two antisymmetric and spectrally separated Fano resonance spectra (Fig. 2d).

### Control of Fano resonances for optical spin switching

Upon revealing the physics behind the Fano-induced optical SAM, we now examine the key parameters for its control in detail. To quantitatively assess the behavior of the system, by following the definition of the Fano parameter^28^ here we define a spin-density Fano parameter *q*_*s*_ ; as the ratio of indirect- to direct-excitation of the resonator, in this case *κ*_*d*_*e*^*iθ*^ and *κ*_*s*_*e*^−*iθ*^ (Eq. (3)). Then, we obtain


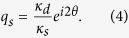


**Figure 4:**
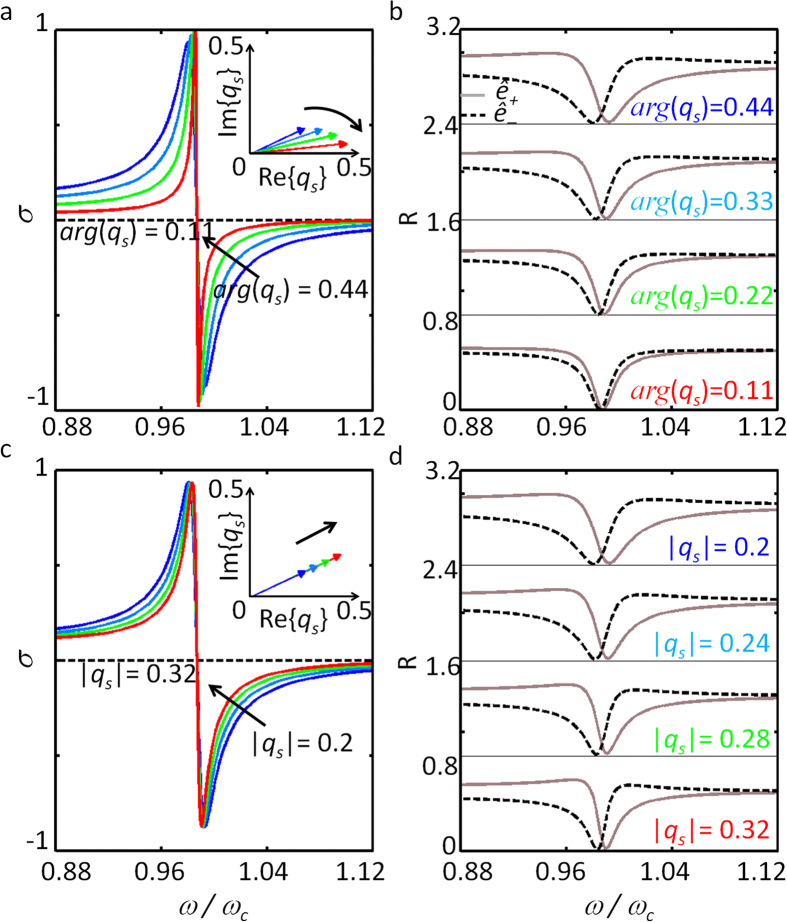
Optical spin-density and reflection spectra as a function of (a,b) chirality i.e. *arg*(*q*_*s*_) = 0.44 to 0.11 and (c,d) birefringence i.e. |*q*_*s*_| = 0.2 to 0.32. In (**b**,**d**) the *ê*_+_ (solid) and *ê*_−_ (dashed) lines represent the states of spin-density *σ* = +1 and −1, respectively.

Figure 4 presents the spin-density spectra as a function of the argument *arg*(*q*_*s*_) = 2*θ* (chirality) and modulus |*q*_*s*_| = *κ*_*d*_/*κ*_*s*_ (birefringence) of the Fano parameter. As observed in Fig. 4a, the bandwidth of the spin density *σ* decreases for smaller *arg*(*q*_*s*_) = 2*θ*, which is associated with the smaller spectral separations between the *ê*_+_ and *ê*_−_ modes (as in Fig. 4b). The spin-density spectra for different |*q*_*s*_| is also plotted in Fig. 4c, showing a smaller bandwidth for larger |*q*_*s*_|, which is again associated with the decrease in the spectral separation between the *ê*_+_ and *ê*_−_ modes (Fig. 4d).

**Figure 5:**
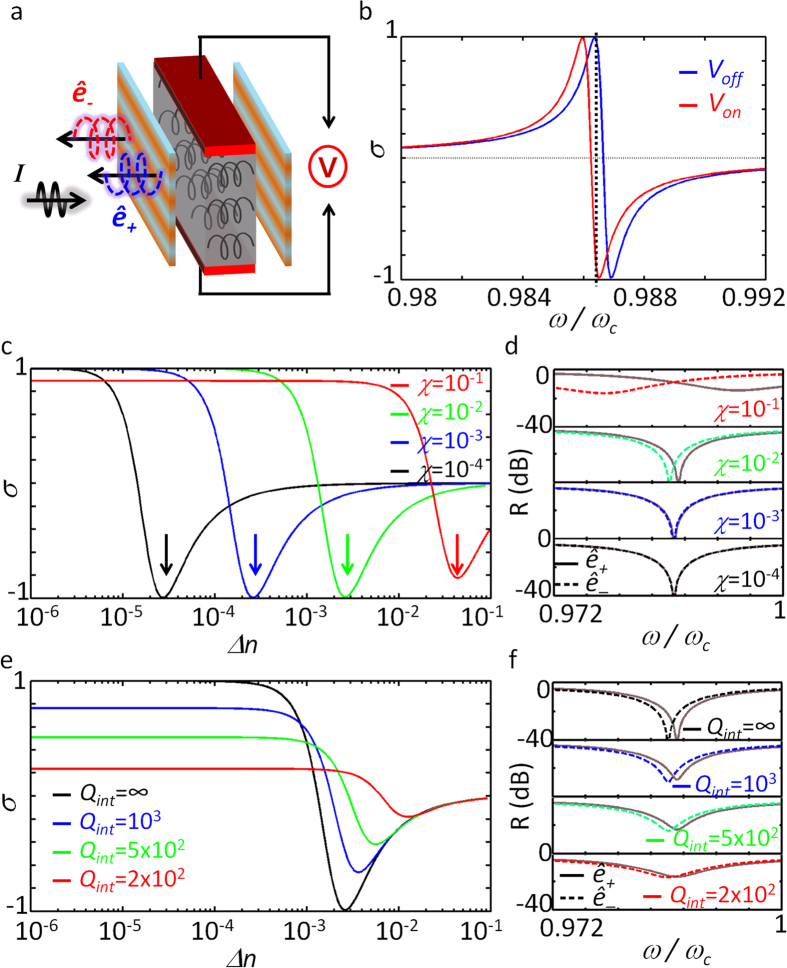
Optical spin switching based on Fano resonances. (**a**) Schematics of the optical spin switching. (**b**) Spin-density spectra without (blue, *V*_*off*_) and with electric bias (red, *V*_*on*_, *Δn* = 0.001). At the frequency 0.987*ω*_*c*_, spin reversal from *σ* = +0.998 (blue) to *σ* = −0.993 (red) is observed. All of the geometrical parameters are the same as those in Fig. 2a, except for *χ* = 0.005. (**c**) The spin density as a function of chirality *χ* (10^−1^ to 10^−4^) and applied *Δn* (10^−6^ to 10^−1^). The magnitude of *Δn*_*R*_ required for the spin reversal for each value of *χ* is marked with arrows. (**d**) Reflectance spectra for *ê*_+_ (solid lines) and *ê*_−_ (dashed lines) at each value of *χ* and *Δn*_*R*_. The effect of material loss for optical spin switching is also presented in terms of (**e**) the spin density and (**f**) reflectance spectra: for different values of intrinsic quality factor of each resonant mode *Q*_*int*_ = 2 × 10^2^ to 10^3^. *χ* = 10^−2^.

Utilizing the sharp transition of the *σ*, reversing its sign from +1 to −1 within *Δω* ~ *ω*_*c*_/250 (Fig. 4), we demonstrate ‘*optical spin switching*’, for the first time to our knowledge. Here we assume spin switching based on the control of *arg*(*q*_*s*_), or equivalently *χ∙L*_eff_. Figure 5a shows the schematics of the suggested device, which has two electrodes connected to the chiral medium for the control of *L*_eff_ via refractive index tuning (e.g., *Δn* ~ 0.008 can be achieved with an ~1 V bias voltage^36^,^37^,^38^). For the change of *Δn* = 10^−3^, in Fig. 5b we show the consequent shift of the spin density spectra, which derives a sharp transition in *σ* ; from *σ* = +0.998 (bias off, blue) to *σ* = −0.993 (bias on, red) at the working frequency *ω* = 0.987*ω*_*c*_ and chirality *χ* = 0.005. As shown in Fig. 5c, even smaller values of *χ* (=10^−1^ to 10^−4^) and smaller tuning of *Δn* (10^−2^ to 10^−5^) for spin switching is also possible, but at the expense of a reduction in reflectance (0.4 to 10^−4^, Fig. 5d). To achieve larger signal strength, chiral metamaterials of lager *χ* (*χ* ~ 1)^4^,^5^,^6^,^27^ combined with background materials with larger index tuning (*Δn* > 10^−3^, such as liquid crystals) could be used with a minor penalty in the purity of the spin. The effect of material loss is also investigated (Fig. 5e,f), by introducing complex-valued resonant frequencies (as in refs 32,33), of *ω*_*h0*_ and *ω*_*v0*_ in Eq. (2): in terms of intrinsic quality factor of each resonant mode *Q*_*int*_ = *Re*[*ω*_*h*,*v0*_]/(2 · *Im*[*ω*_*h*,*v0*_]). Due to the spectral broadening and imperfect critical coupling from material loss, the purity of the spin density decreases (Fig. 5e), yet with the overall increase in its reflectance (Fig. 5f).

## Discussion

In this work, we propose a new pathway for the nonmagnetic achievement of optical spin angular momentum based on the spin-dependent separation of Fano resonance spectra. By developing a spin-form temporal CMT for the chiral resonator, we unveil the origin of the spin-dependent antisymmetric Fano resonance in perfect agreement with the scattering matrix calculations. A spin-density Fano parameter is derived to identify key parameters for quantitative control of the optical SAM in the spectral domain. Based on the spectrally-sensitive characteristics of the Fano resonance, ‘optical spin switching’ is proposed for the first time, with experimentally accessible parameter values. From an order of magnitude reduction of material chirality required for optical SAM generation (Fig. 5c–f), we can also envisage the applications for bio-chemical sensing: the sensing of organic helical structures (very weak chirality from the displacement current) without any metallic inclusions (strong chirality from the conduction current). In Supplementary Information, we provided the real implementation of Fano- induced spectral separation of optical SAM using highly-birefringent mirrors (Supplementary Note 1, see Supplementary Fig. 2 for their fabrication process), especially analyzing the property of birefringent mirrors in IR and THz regimes (Supplementary Note 2). Our results, which are based on ‘conservative (lossless)’ optical elements fundamentally distinctive from non-conservative circular dichroism, not only derive high-*Q* responses from the Hermiticity, but also allow excellent purity in spin density (*P*_*RCP*_/*P*_*LCP*_ < −15 dB in Figs 2 and 4, with practical parameters both in IR and THz regimes) comparable to the previous record in circular dichroism (*P*_*RCP*_/*P*_*LCP*_ = −1.5 dB in the IR range^39^, and −25 dB in the microwave range^40^).

## Additional Information

**How to cite this article**: Piao, X. *et al*. Spectral separation of optical spin based on antisymmetric Fano resonances. *Sci. Rep*. **5**, 16585; doi: 10.1038/srep16585 (2015).

## Supplementary Material

Supplementary Information

## Figures and Tables

**Figure 1:**
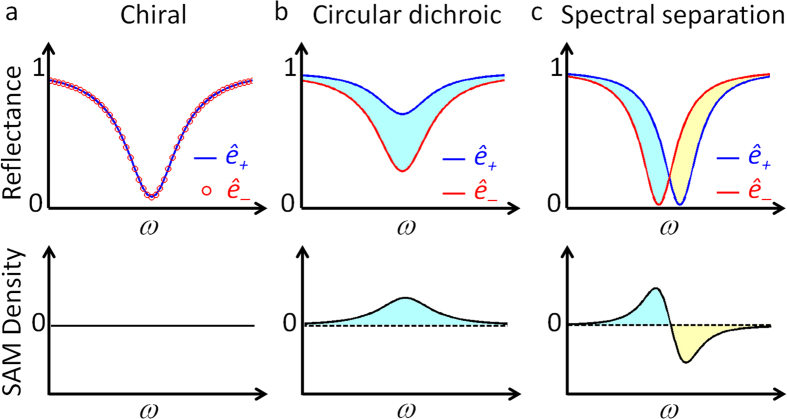
A schematic of reflection spectra for linear polarization and corresponding density of optical SAM; from (a) chiral material, (b) circular dichroic material, and (c) spin-dependent spectral separation system. *ê*_+_ (blue) and *ê*_−_ (red) represent SAM of +1 and −1.
