# Supplementary material for: Spectral separation of optical spin based on antisymmetric Fano resonances
Source: Sci Rep. 2015 Nov 12;5:16585. doi: 10.1038/srep16585 (PMC4642317; doi:10.1038/srep16585)
Supplement: Supplementary Information [file srep16585-s1.pdf]

Supplementary Information

Spectral separation of optical spin based on antisymmetric Fano resonances

Xianji Piao, Sunkyu Yu, Jiho Hong, and Namkyoo Park*

Photonic Systems Laboratory, School of EECS, Seoul National University, Seoul 151-744, Korea

**E-mail address for correspondence: nkpark@snu.ac.kr*

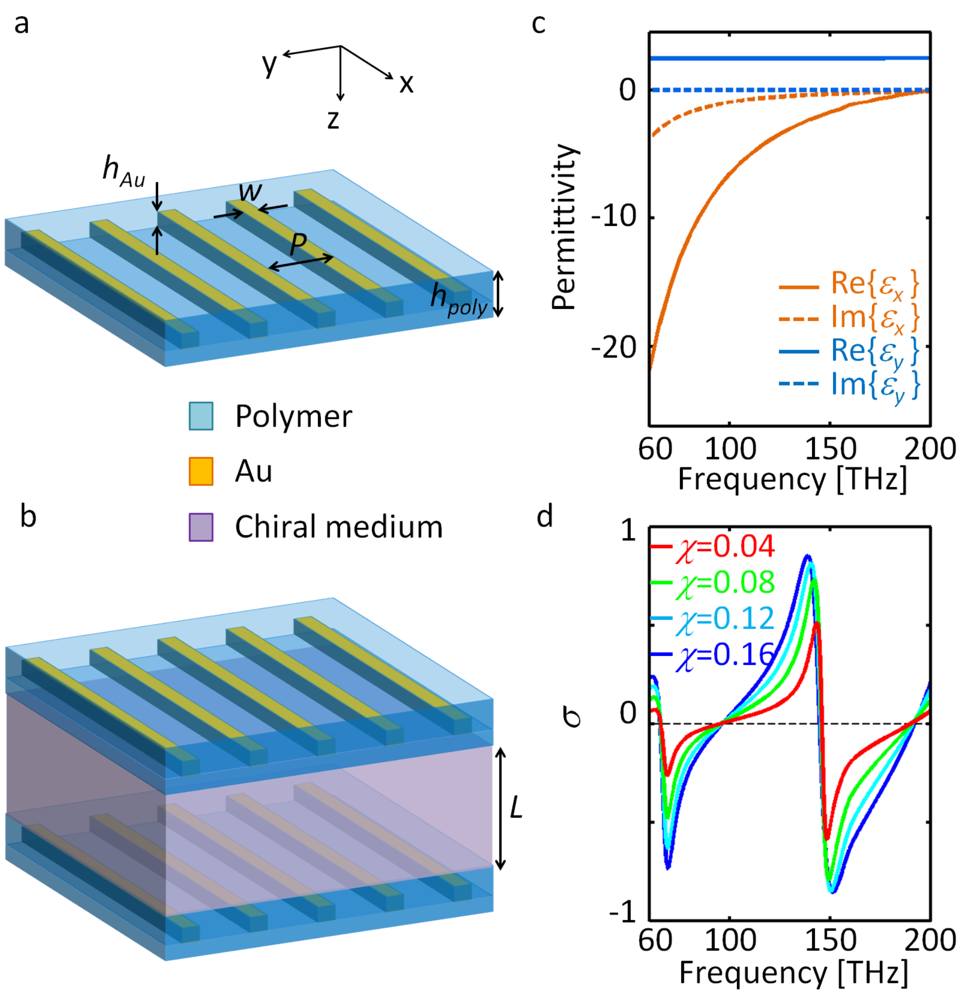

Supplementary Figure 1. Implementation example of Fano-resonant spin separation. Schematic view of the **a,** indefinite metamaterial mirror, composed of a layer of gold grating (h_{poly} = 100 nm, h_{Au} = 40 nm, w = 40 nm, P = 400 nm) embedded in the polyimide; and **b,** Structure of the chiral resonator **c,** Effective permittivity values of the indefinite metamaterial mirror calculated from the 3D Finite Element Method (FEM) simulations. Material values of *Au* and polyimide are taken from Supplementary Refs 2 and 3. **d,** Spin density spectra for different values of χ (0.04 to 0.16), with the inclusion of realistic material parameters for the birefringent mirror. Scattering matrix method has been used for the calculation of spin density.

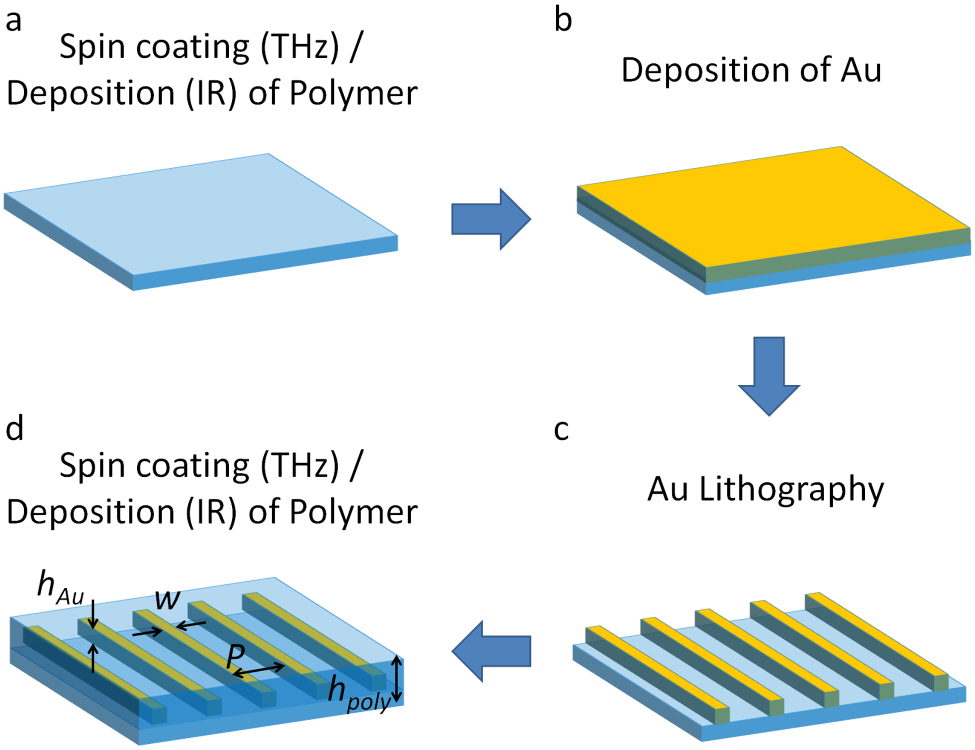

Supplementary Figure 2. Fabrication process of an indefinite birefringent mirror. a, Deposition (CVD or ALD for the IR regime, for tens of nm thickness) or spin coating (for THz regime with μm thickness) of polymer. **b,** Metal deposition (e-beam or thermal evaporation, for tens of nm thickness). The sub-nm titanium adhesion layer is used in general. **c,** Lithography for the formation of a gold grating (tens of nm for the IR regime, or hundreds of nm for the THz regime, which are easily achieved with conventional lithography). **d,** Coating / deposition of polymer top layer. CMP may be required optionally.

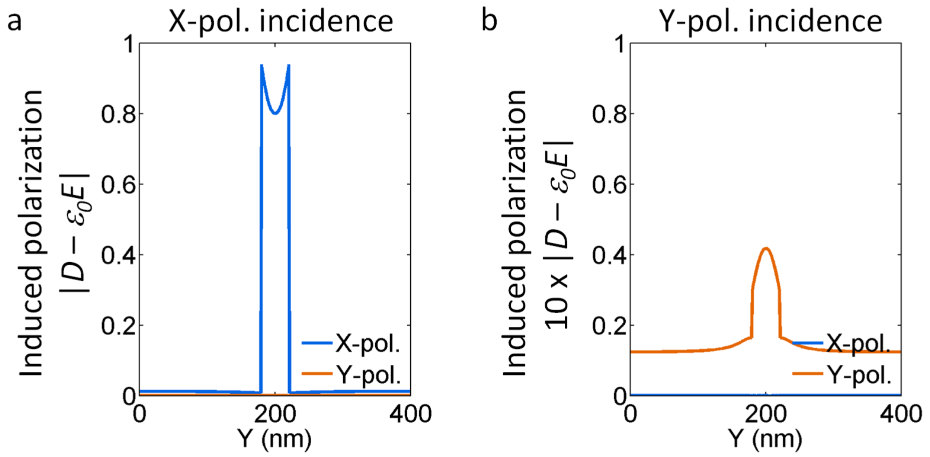

Supplementary Figure 3. The strength of induced polarizations (*D* - *ε_0E*) for **a,** *x*-polarized and **b,** *y*-polarized incidences, at 150 THz. h_{Au} = 40 nm, h_{poly} = 100 nm. w = 40 nm, and P = 400 nm. The permittivity of the gold is obtained from the Palik[2], and the polymer has the permittivity of $\varepsilon_{poly} \sim 2.25$ in the IR regime[3]. Calculations have been made with the 3D finite element method of COMSOL Multiphysics.

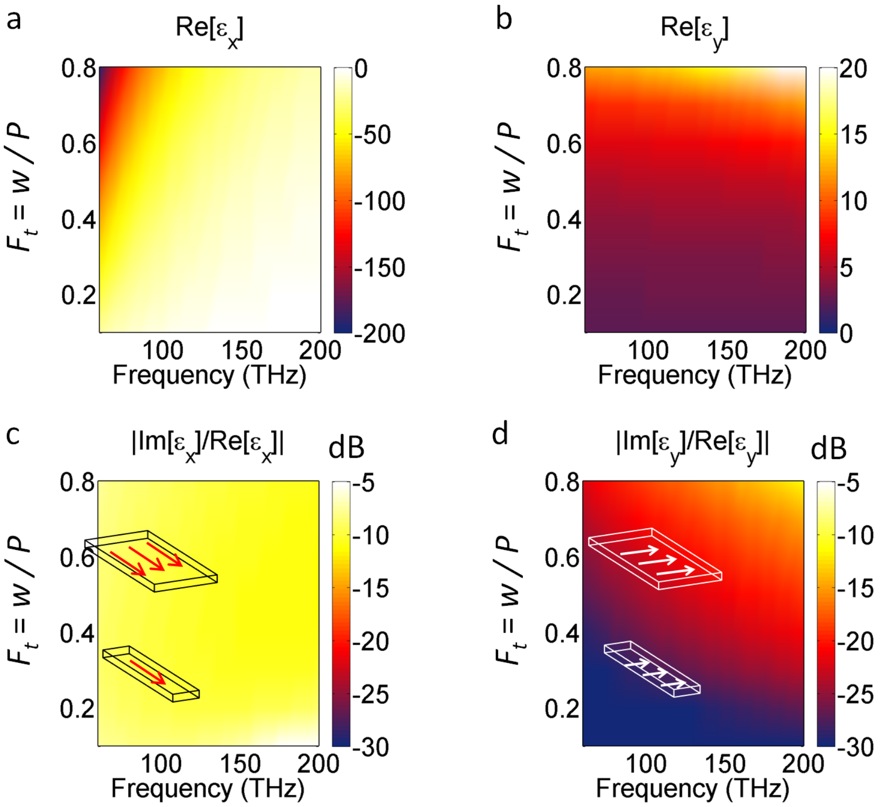

Supplementary Figure 4. Anisotropic permittivity and loss of the birefringent mirror (Supplementary Fig. 1a) in the IR regime, as a function of frequency and transverse filling ratio F_t; along x- (parallel to metallic gratings) and y- (perpendicular to metallic gratings) axis: **a,** Re[ε_x], **b,** Re[ε_y], **c,** Im[ε_x] / Re[ε_x], and **d,** Im[ε_y] / Re[ε_y]. h_{Au} = 40 nm, h_{poly} = 100 nm. w is the width of the metal, and P = 400 nm is the period of the grating. The permittivity of gold is obtained from the Palik[2], and the polymer has the permittivity of ε_{poly} ~ 2.25 in the IR regime[3]. All the calculations are based on 3D FEM.

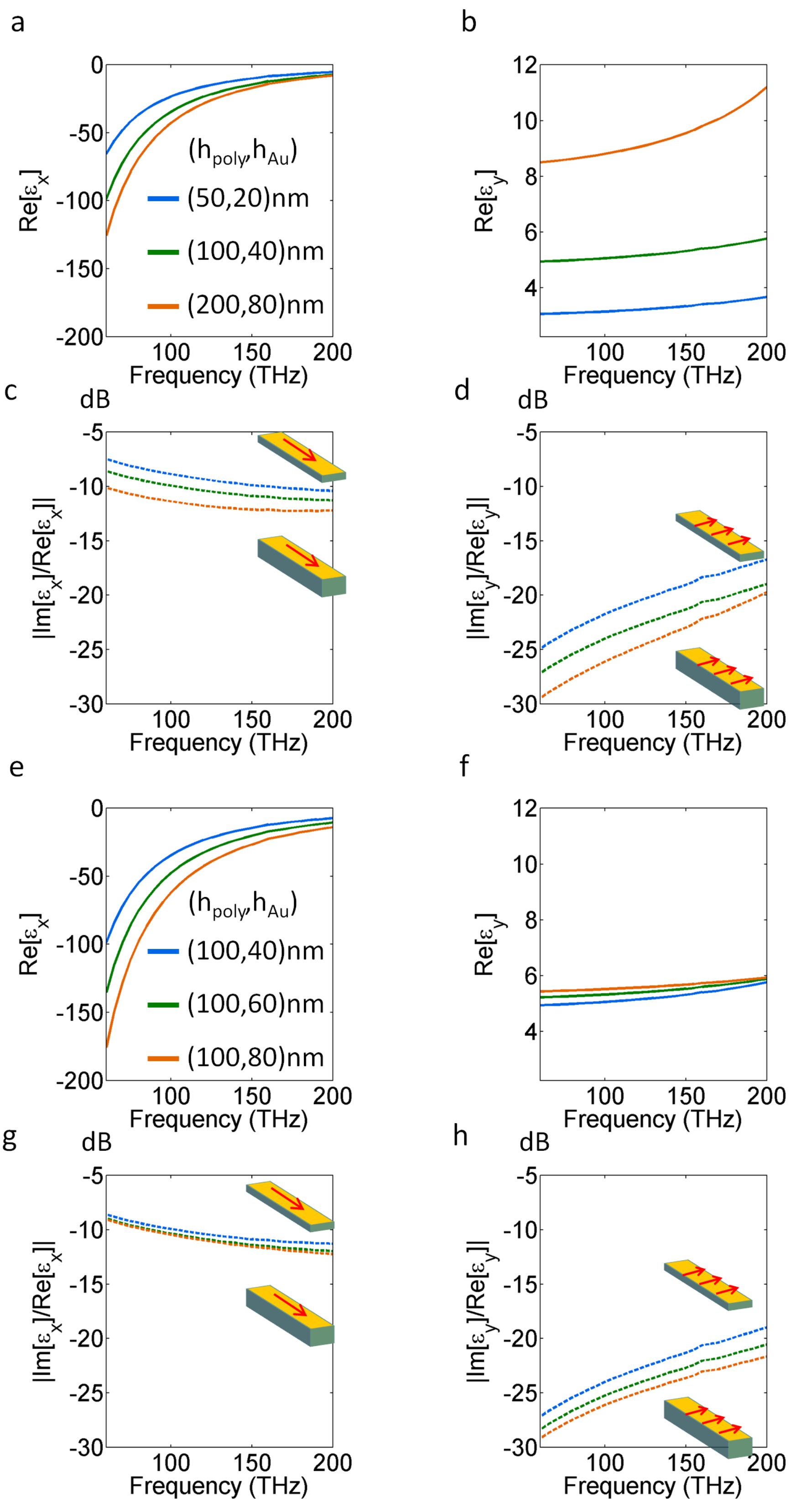

Supplementary Figure 5. The effect of longitudinal structural parameters: (**a-d**) the mirror thickness h_{poly} maintaining h_{Au} / h_{poly} = 0.4, and (**e-h**) the thickness of metal layer h_{Au} for h_{poly} = 100 nm. In all the cases P = 400 nm and w = 200 nm.

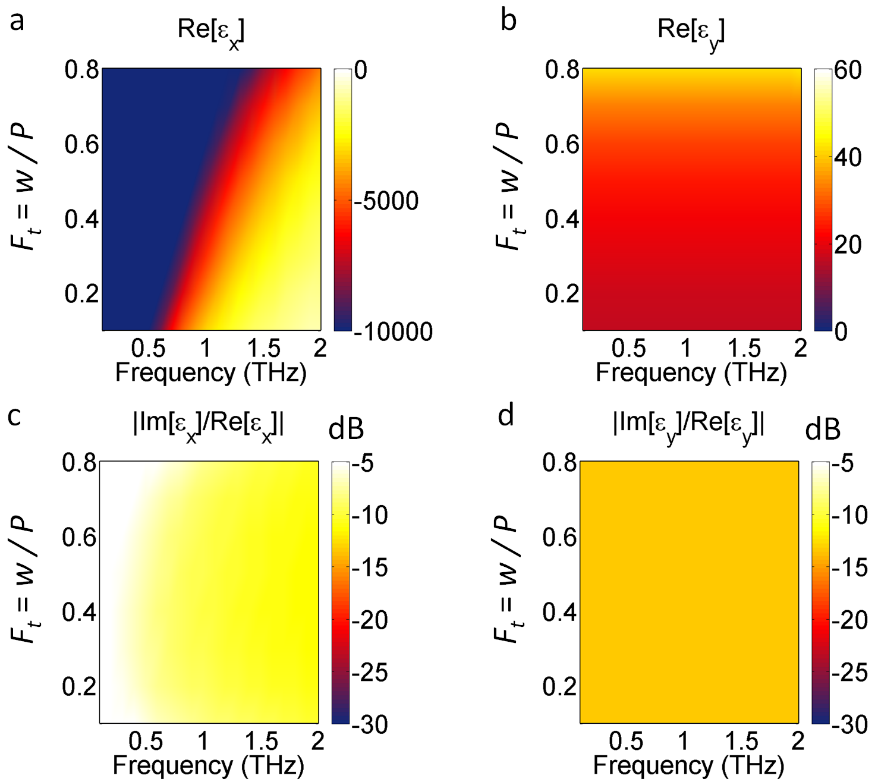

Supplementary Figure 6. Anisotropic permittivity and loss of the birefringent mirror (Supplementary Fig. 1a) in the THz regime, as a function of frequency and transverse filling ratio F_t; along *x*- and *y*- axis: **a,** Re[ε_x], **b,** Re[ε_y], **c,** Im[ε_x] / Re[ε_x], and **d,** Im[ε_y] / Re[ε_y]. h_{Au} = 100 nm, h_{poly} = 2500 nm. P = 4 μm. The permittivity of gold is obtained from the Drude model, and the polymer has the permittivity of $\varepsilon_{poly} \sim 3.24 - 0.14i$ in the THz regime[7].

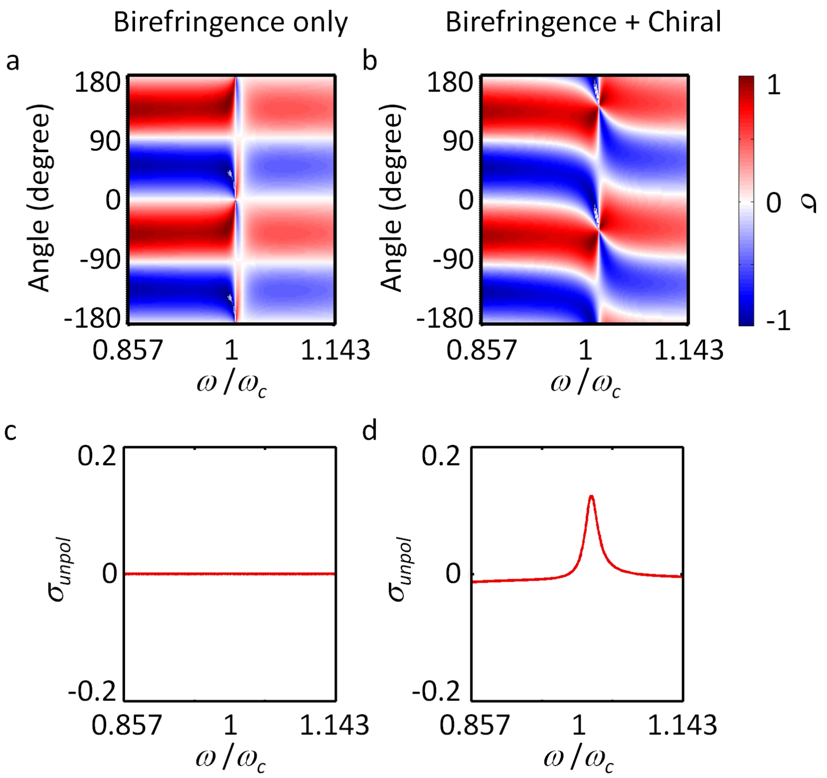

Supplementary Figure 7. Density of optical SAM in reflection spectra for the state of linear polarizations: (a,c) non-chiral core medium ($\chi = 0$) and (b,d) chiral core medium ($\chi \neq 0$). (a,b) Dependency on the state of linear polarizations. (c,d) Density spectra under the unpolarized incidence (σ_{unpol}). For all the cases, the birefringent mirror has $\varepsilon_x = -50$, $\varepsilon_y = 12.25$, and $D = 0.028\lambda_c$, and the core material of the Fabry-Perot resonator has $\varepsilon_c = 2.25$, $L = 0.28\lambda_c$ where $\lambda_c = 2\pi c / \omega_c$, ($\sigma = +1$ and -1 each represents $\hat{e}_+$ and $\hat{e}_-$).

Supplementary Note 1. Real implementation using indefinite birefringent mirror

Supplementary Figure 1 presents the implementation example of Fano-resonant spin separation using typical material and structural parameters supported by current fabrication technologies. Specifically, we show an indefinite ($\varepsilon_x < 0$ and $\varepsilon_y > 0$) birefringent mirror composed of metal grating embedded in a polymer film (Supplementary Figs 1a,1b. see also Supplementary Fig. 2 for the fabrication process). It is noted that the control of effective material parameters can be achieved with the change of structural parameters, both in IR and THz regimes (see Supplementary Note 2 for details). Supplementary Figure 1c shows the effective anisotropic permittivity of the film, calculated with[1] for the IR regime operation; employing the gold (*Au*, ref. 2) grating in a polyimide[3] film (h_{poly} = 100 nm, h_{Au} = 40 nm, w = 40 nm, and P = 400 nm). Even with the inclusion of realistic material loss and spectral dispersion, sharp transition in the spin density spectra are successfully achieved (Supplementary Fig. 1d), where its bandwidth can be controlled with different values of chirality (same χ values as in Fig. 4a in the main manuscript).

For the practical implementation of Fano-resonant spin separation structures, we consider following requirements for highly-birefringent mirrors; (1) a 'flat' film-type mirror, for the serial deposition of a bottom mirror, a chiral medium, and a top mirror; (2) the indefinite ($\varepsilon_x < 0$ and $\varepsilon_y > 0$) non-resonant effective permittivity tensor of the mirror; (3) tunability of the effective material parameters for the control of the degree of spin separation. Under these requirements, here we employ the non-resonant metallic grating embedded in a dielectric film (Supplementary Fig. 1a).

Supplementary Figures 2 shows the fabrication process of the suggested indefinite mirror. Firstly, the dielectric layer can be constructed for example, by using the spin coating method with soluble polymers[3] to achieve μm-scale mirror thickness (~λ_0/100) for the THz operation. For the IR or visible regime applications, thin-film deposition methods for insoluble dielectrics[4] or soluble polymers[5] could be used to achieve tens of nm mirror

thickness ($\sim\lambda_0/100$) (e.g. based on the chemical vapor deposition (CVD)[4] or atomic layer deposition (ALD)[5] both guaranteeing sub-nm-scale precision). A gold layer can then be overlaid using electron-beam or thermal evaporation (Supplementary Fig. 2b). After the lithography or imprinting process for the formation of grating pattern (Supplementary Fig. 2c), the shielding top dielectric is overlaid (Supplementary Fig. 2d) using the same method as in Supplementary Fig. 2a, completing the flat birefringent film. We note that, in contrast to the spin coating method, thin-film deposition may result in the surface roughness (due to the gold grating pattern, ~ tens of nm in the IR regime), which however can be removed completely using the chemical-mechanical polishing (CMP) technique offering the sub-nm flatness[6].

Supplementary Note 2. Material properties of indefinite birefringent mirrors in IR and THz regimes

In this Supplementary Note 2, we investigate the effective material parameters[1] of the mirror structure in Supplementary Fig. 1a and 2, using the 3D finite element method of COMSOL Multiphysics. We focus on two different frequency ranges: IR regime (60 to 200 THz) and THz (0.1 to 2.0 THz) regime.

At first we show the induced polarization inside the grating mirror under *x*- or *y*-polarized plane wave incidences (Supplementary Fig. 3, measured at the mid-plane of the indefinite mirror). For the incidence of *x*-polarized (TM-like) light, the formation of strong localized wire plasmons are evident at the Au-polyimide interfaces (Supplementary Fig. 3a), in contrast to the case of a *y*-polarized (TE-like) incidence (Supplementary Fig. 3b) which shows much weaker (1/500 intensity compared to *x*-polarized incidence) and spatially-spread induced polarizations into the polyimide regions. This result well justifies our expectation of the indefinite mirror design in Supplementary Fig. 1, having metallic *x*-axis component and dielectric *y*-axis component effective permittivity.

Supplementary Figure 4 shows the anisotropic permittivity of the birefringent mirror in the IR regime, as a function of operation frequency and transverse filling ratio F_t (width of metal / grating period = w / P) at *fixed* h_{Au} = 40 nm, h_{poly} = 100 nm, and P = 400 nm. The real part of ε_x, which originates from the plasmonic polarization in parallel to the metallic wires, has large negative value, varying rapidly with the increase of F_t (Re[ε_x] = –200 to –1 for $0.1 \leq F_t \leq 0.7$, Supplementary Fig. 4a). To compare, Re[ε_y] is relatively stable against the change of metal width (Re[ε_y] = 2.5 to 10 for $0.1 \leq F_t \leq 0.7$, Supplementary Fig. 4b) exhibiting the expected dielectric behavior. We also note that, because the loss part of permittivity does originate only from the induced current density in the metal region, Im[ε_x] is highly-stable (~ –10 dB, Supplementary Fig. 4c); meanwhile, Im[ε_y] varies significantly (–30 to –10 dB, Supplementary Fig. 4d) dependent on F_t.

The effective permittivity can also be controlled with the variation of h_{poly} or h_{Au} (Supplementary Fig. 5). Identical to the case of Supplementary Fig. 4, the absolute value of the real part of permittivity (Supplementary Fig. 5a,5b,5e,5f) increases when the amount of metal increases. In contrast to the case of F_t control, the increase of h_{Au} results in the reduced loss. This effect originates from the decrease of the Ohmic resistance due to larger metal thickness.

Supplementary Figure 6 shows the result of frequency-dependent birefringent permittivity in the THz regime, which provides similar features with Supplementary Fig. 4, but with much larger magnitude of ε_x and ε_y, from the PEC-like property of metal in the THz regime. As can be seen, at F_t = 0.1 (w = 400 nm and P = 4 μm), wide spectral range is guaranteed near 1.5 THz, also providing reasonable effective parameters (Re[ε_x] ~ -10^3 and Re[ε_y] ~ 15, with loss parameters of –10 dB and –15 dB, respectively).

Supplementary Note 3. Fano-resonant spin separation for arbitrary linear polarizations

Here we investigate the Fano-resonant spin separation for arbitrary linear polarizations, using scattering matrix calculations. Supplementary Figures 7a and 7b show the optical SAM density (σ) of the reflection beam as a function of the frequency and the state of linear polarizations. We can see that the chiral resonator system not only derives Fano spectral asymmetry for arbitrary polarization angle (Fig. 7b vs. 7a), but also achieve the non-zero SAM (σ_{unpol}) for the unpolarized incident beam (Fig. 7d vs. 7c). Such a difference originates from the chiral material, which realizes the Fano mixing of narrow- (x-axis) and broad- (y-axis) scattering paths from its spin-based eigenvectors with different effective indices.

Supplementary References

1. Smith, D. R., Schultz, S., Markoš, P. & Soukoulis, C. M. Determination of effective permittivity and permeability of metamaterials from reflection and transmission coefficients. Phys. Rev. B **65**, 195104 (2002).

2. Palik, E. D. *Handbook of Optical Constants of Solids* (New York, Academic, 1985).

3. Saito, M., Gojo, T., Kato, Y. & Miyagi, M. Optical constants of polymer coatings in the infrared, Infrared Phys. Technol. **36**, 1125-1129 (1995).

4. Klaus, J. W. & George, S. M. SiO_2 Chemical Vapor Deposition at Room Temperature Using $SiCl_4$ and H_2O with an NH_3 Catalyst. J. Electrochem. Soc. **147**, 2658-2664 (2000).

5. Grunze M. & Lamb, R. N. Preparation and adhesion of ultrathin polyimide films on polycrystalline silver. Chem. Phys. Lett. **133**, 283-287 (1987).

6. Chen, W. C., Lin, S. C., Dai, B. T. & Tsai, M. S. Chemical Mechanical Polishing of Low-Dielectric-Constant Polymers: Hydrogen Silsesquioxane and Methyl Silsesquioxane. J. Electrochem. Soc. **146**, 3004-3008 (1999).

7. Choi, M. et al. A terahertz metamaterial with unnaturally high refractive index. Nature **470**, 369-373 (2011).
